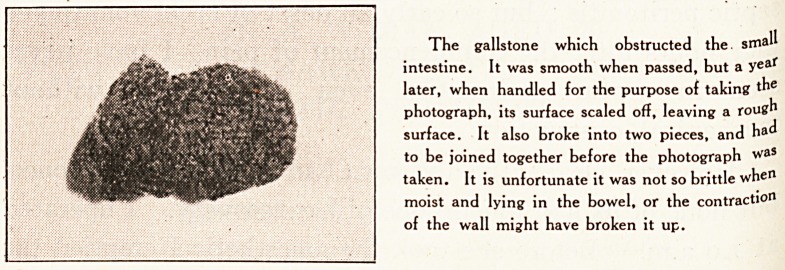# Recovery after Mechanical Obstruction Due to the Passage of a Gallstone through the Intestine, with Septic Peritonitis

**Published:** 1924-07

**Authors:** Charles A. Morton

**Affiliations:** Professor of Surgery in the University of Bristol; Consulting Surgeon to the General Hospital and to the Children's Hospital, Examiner in Surgery in the University of Birmingham


### A CASE OF RECOVERY AFTER MECHANICAL OBSTRUCTION DUE TO THE PASSAGE OF A GALLSTONE THROUGH THE INTESTINE, COMBINED WITH A DIFFUSE SEPTIC PERITONITIS, IN A WOMAN AGED 70; WITH SOME REMARKS ON THE TREATMENT OF SEPTIC PERITONITIS.

BY

CHARLES A. MORTON, F.R.C.S.,

*Professor of Surgery in the University of Bristol;*
*Consulting Surgeon to the General Hospital and to the Children's Hospital;*
*Examiner in Surgery in the University of Birmingham.*

---

MRS. G., aged 70, was seen in consultation with Dr. Perrott, of Kingswood, at 11.0 p.m. on Tuesday, March 20th, 1923. The history was that at 4.0 a.m. that morning she had fallen on the floor, and that very soon after severe pain had commenced in the upper abdomen, extending down the sides. She was able to go downstairs about breakfast-time, but the pain was so severe that by 11.0 a.m. she had to return to bed again, and the severity of the pain persisted. Vomiting began at 8.0 a.m., and by 2.0 p.m. it had become fæcal and was very frequent. There was a history of epigastric pain and vomiting for some years.

When I saw her she was calling out with the severity of the pain in her abdomen, and this seemed to be of a diffuse character. The abdomen was markedly and generally distended and universally tender, with areas of resonance and dullness. There was no rigidity. Her pulse was 100, and remarkably good for the serious condition she was in. I have no note of her temperature, but I remember it was not definitely raised. The extreme tenderness of the abdomen certainly suggested peritonitis rather than a mechanical obstruction, and as I have seen such marked and persistent fæcal vomiting in a general septic peritonitis, without any mechanical obstruction other than the kinking of the distended coils, I thought it might be an acute general septic peritonitis; but so early an onset of fæcal vomiting—four hours after the commencement of pain—I have never seen in septic peritonitis, even if general, without mechanical obstruction.

However, clearly the abdomen had to be opened at once, but none of us had much hope of her recovery. I operated at 1.0 a.m. Before she took the anæsthetic I emptied the stomach of a considerable quantity of fæcal fluid, and then washed it out. On opening the abdomen by an incision close to the middle line, through the right rectus below the umbilicus, I evacuated a large quantity of pus (not offensive), and on introducing my hand and pushing aside some distended coils, evacuated more pus from the pelvis. Red, distended coils of intestine, with patches of lymph exudation on them, presented. The peritonitis was widely diffused in the lower abdomen, but it was not possible to say to what extent it invaded the upper abdomen. It was clearly not due to disease of the appendix or fallopian tubes, and indeed no cause could be discovered in the lower abdomen. I passed my hand into the upper abdomen and found a mass of omentum fixed in the region of the gall-bladder, which was lost in it. I found a large gallstone (shown below) in a coil of small intestine, but though tightly gripped by the wall of the bowel, I could with difficulty move it along. The question arose whether I should remove it, but in the presence of septic peritonitis, which would have seriously interfered with the healing of the intestinal incision, and as it was not absolutely fixed, I decided not to do so. I swabbed out the lower abdomen and pelvis, and inserted a large split rubber drainage tube into Douglas's pouch, and closed the abdominal wall around it. Her pulse was 140 at the end of the operation, but to our great surprise and satisfaction was as strong as at the commencement.

The gallstone which obstructed the small intestine. It was smooth when passed, but a year later, when handled for the purpose of taking the photograph, its surface scaled off, leaving a rough surface. It also broke into two pieces, and had to be joined together before the photograph was taken. It is unfortunate it was not so brittle when moist and lying in the bowel, or the contraction of the wall might have broken it up.

Subcutaneous saline infusion was started as soon as she was back in bed, and $\frac{1}{6}$ of a gr. of morphine was given to relieve the pain, which was not, however, as great as before the operation. In the early morning the fæcal vomiting recurred and was frequent and copious, and I had to wash out the stomach; nevertheless, her pulse had fallen to 104 and was still of fairly good strength. She absorbed eight pints of saline in the first twenty-four hours, and as her pulse kept so good I did not continue it. The fæcal character of the vomiting ceased about twelve hours after the operation, and then the vomit consisted of a dark brown fluid, but this was not offensive.

On Thursday the 22nd (the day following the operation)

the only pain she had was in the upper abdomen, such as she had been subject to for some time. The vomiting had ceased. She was only fed by nutrient enemata, all of which she retained. Hot turpentine enemata were given every four hours, but only a stain of fæcal matter resulted, and the distension of the abdomen persisted, but in the night she had two loose, spontaneous actions. There was a very considerable discharge of foul serum from the drainage tube at the end of the first twenty-four hours. I then removed the tube.

On Friday the 23rd she began peptonised milk by mouth, but vomiting recurred, and the vomit was of a dark brown colour, and on two occasions (which were not consecutive) was again fæcal.

On Saturday the 24th, as there had been no further action of the bowels, even with the continued administration of hot turpentine enemata, and of a dose of calomel (though a little flatus had been spontaneously passed), and the abdominal distension persisted, I gave her an intramuscular injection of pituitary extract, 1 c.c. This very quickly produced some sharp attacks of abdominal pain, and within half an hour of the administration she passed half a pint of fæces and some flatus. This was followed by a marked decrease of the abdominal distension and a cessation of the vomiting. She then had four spontaneous actions and passed more flatus. But even after this the abdominal distension again increased to a marked extent, and another injection of pituitary extract, this time $1\frac{1}{2}$ c.c., was given and was repeated every six hours, and every injection was very quickly followed by a loose action. She had much griping pain in the abdomen, both with and without these injections, and with this pain I could make out distension and hardening of coils in the lower abdomen. This was on Monday the 26th. She had only recurrences of the vomiting at considerable intervals, and took all her nourishment by mouth.

On Wednesday the 28th, after a small spontaneous loose action, she had much distress in the anal region, and the Sister helped her to pass a gallstone (see page 128), which measured $1\frac{1}{4}$ inches by $\frac{3}{4}$ of an inch. She then had seven copious loose actions within six hours, and the hardening of coils ceased, though there was still moderate general distension. After the removal of the drainage tube there had been very little discharge from the track, until this day (Wednesday the 28th), when considerable offensive purulent discharge took place, and I re-introduced a small drainage tube. The spontaneous loose actions continued, and by Friday the 30th the abdominal distension had subsided. At no time after the operation was there any pyrexia, and her pulse kept about 104 to 112, and did not fail in strength. From this time her progress was uninterrupted and she returned home in May. Since that time until the last few weeks (April, 1924) she has been very well, and by taking an aperient has kept the bowels acting satisfactorily. She has been free from the old dyspeptic symptoms. But a few weeks ago she began to suffer from some chest trouble, which laid her up in bed, and while suffering in this way, on March 24th of this year, she complained of a slight pain in the lower abdomen on coughing and sitting up in bed, and a swelling was discovered in the right lower abdomen. Shortly after, an abscess formed in the overlying abdominal wall and discharged. All discharge has now ceased, but I hear there is still some deeper swelling. The patient is, however, able to be up again.

It is, of course, well recognised that in obstruction from a gallstone in the intestine the stone may be passed, with recovery of the patient, so that the passage of the stone in this case was not very remarkable, but recovery from such a condition, combined with a diffuse septic peritonitis, accompanied by marked and persistent fæcal vomiting, seems to warrant its record.

The cause of the peritonitis, and the reason why the mechanical obstruction and the peritonitis started simultaneously, is an interesting question. I believe the answer to be that probably for some time the gallstone had caused, by ulceration, an opening between the adherent gall-bladder and the duodenum, and that the anastomosis was surrounded, or at any rate partly bounded, by a mass of adherent omentum; and in her fall she probably pushed the gallstone through the opening into the duodenum, and at the same time tore the protective adhesions to the omentum, thus laying open the sac in which the stone lay to the general peritoneal cavity. If her condition at the time of the operation had not been so serious I should have explored the gall-bladder region by a high incision, but I did not think she was in a condition to bear any further operative procedure. The passage of the gallstone seems to have cured her of the dyspeptic symptoms from which she had suffered for some years.

The very frequent and persistent vomiting in this case was what very frequently occurs in this form of intestinal obstruction. Treves in his classical work on intestinal obstruction wrote, "I know of no form of intestinal obstruction in which the vomiting is more incessant, more obstinate, and more copious than it is in cases in which the upper jejunum is blocked by a gallstone." And he also says the vomit early becomes fæcal, and quotes Schüller that it was so in 77 cases out of 120. Of course, in this case the vomiting was probably due to both the mechanical obstruction and peritonitis, but the fact that it became fæcal so very early in the case seems to me to indicate that it was mainly due to the mechanical obstruction. And the fact that it persisted exactly up to the time the stone was passed (and it was probably passed very soon after passing through the ileo-cæcal valve) seems to me also to point to the mechanical obstruction as, at any rate, the cause of its persistence.

I cannot understand the objection which some surgeons have at the present day to drainage of the abdomen in cases of diffuse and general peritonitis. My only regret about the treatment of this case is that I removed the drainage tube as early as I did, because I thought that its tract must by that time be shut off from the general peritoneal cavity by adhesions. Yet there was a discharge of a considerable quantity of offensive pus from the drainage tube track some days later. Would any surgeon think of not draining an acute abscess? Then why should not a collection of pus in the abdominal cavity, the toxins from which will, when absorbed, seriously affect the patient, be drained? I admit that the drainage track soon gets shut off by adhesion and does not drain the whole peritoneal cavity for long; but this early drainage is most important, and the drainage tube will continue to drain the pelvis and the loin pouches for some time. The fact that in this case the copious discharge of pus, which took place along its track even after the drainage tube had been removed for twenty-four hours, shows that such drainage may occur.

And I do not like the modern innovation of the use of a strand or roll of soft rubber tissue, instead of a rubber drainage tube. I do not believe the latter inflicts any serious harm on the gut, whereas in a roll of soft rubber tissue the lumen is probably very soon obliterated by the pressure of surrounding distended coils, and such pressure will block the passage through which a mere strand of soft rubber tissue passes. Of course, the rubber tube must never have holes cut in it, for into these holes omentum may pass, and the strands become strangulated and swollen and thus anchor the tube. But a split tube forms the best drain, I think, we can employ.

Neither can I see sufficient reason for abandoning the practice of as thoroughly removing pus at the time of the operation as possible, either by swabbing or even flushing. I admit that unless the peritonitis is general flushing is contra-indicated, for it may disseminate the pus into regions of the abdomen not yet infected; but if the peritonitis is general, and if it can be carried out without turning distended coils out of the abdomen (a very shock-producing procedure), *i.e.* if the coils are not too distended to allow of the free passage of the fluid between them, then I believe it is the best method we can employ. Those who object to it base their objection on two statements. One is that such irrigation damages the endothelium of the peritoneum, and therefore increases the liability to absorption of toxins, and the other is that flushing will remove the phagocytes. I do not believe that flushing with normal saline damages the endothelium of the peritoneum, for the phagocytes only exist on the surface of the peritoneum and as soon as the leucocytes mix with the pus they are rendered inert as phagocytes; and surely it is more important to remove as thoroughly as possible pus containing a considerable quantity of deadly toxin than to preserve a layer of phagocytes on the surface of the peritoneum. Irrigating the peritoneum with hot saline is not a shock-producing procedure. I have known the pulse actually improve while it was being carried out. If there is any extravasation of irritating fluid, such as stomach contents, even those opposed to irrigation in generalised peritonitis seem inclined to adopt it. However, unless the peritonitis or, at any rate, extravasation of irritating fluid is general it is better not to flush, but in such cases it seems to me a decided advantage to swab up all the pus. This should be done very gently, so as not to remove the lymph exudation from the peritoneum (for this prevents toxin absorption), and so as not to damage the surface of the peritoneum where not protected by this lymph. I feel sure pools of pus in the pelvis and loin pouches and elsewhere can with advantage be thus evacuated. It must be a decided advantage to get rid of all this toxin-laden fluid, even though more is exuded, especially if we are wise enough to drain these pouches.

It is, of course, most desirable in the operative treatment of septic peritonitis to remove the cause—a diseased appendix or suppurating tube, or close a perforated gastric ulcer; but in some cases the patient may be too bad to allow of this, and in these cases Murphy of Chicago, some years ago, showed us how life might be saved in these desperate cases by the simple proceeding of introducing a large drainage tube into the pelvis through a small supra-pubic incision.

## Figures and Tables

**Figure f1:**